# Nitric Oxide in the Control of the *in vitro* Proliferation and Differentiation of Human Hematopoietic Stem and Progenitor Cells

**DOI:** 10.3389/fcell.2020.610369

**Published:** 2021-02-09

**Authors:** Julia Hümmer, Saskia Kraus, Katharina Brändle, Cornelia Lee-Thedieck

**Affiliations:** ^1^Institute of Functional Interfaces, Karlsruhe Institute of Technology, Eggenstein-Leopoldshafen, Germany; ^2^Institute of Cell Biology and Biophysics, Leibniz University Hannover, Hannover, Germany

**Keywords:** nitric oxide, hematopoietic stem cells, proliferation, differentiation, gasotransmitter, ROS/RNS

## Abstract

Hematopoietic stem and progenitor cell (HSPC) transplantation is the best-studied cellular therapy and successful *in vitro* control of HSPCs has wide clinical implications. Nitric oxide (NO) is a central signaling molecule *in vivo* and has been implicated in HSPC mobilization to the blood stream in mice. The influence of NO on HSPC behavior *in vitro* is, however, largely obscure due to the variety of employed cell types, NO administration systems, and used concentration ranges in the literature. Additionally, most studies are based on murine cells, which do not necessarily mimic human HSPC behavior. Thus, the aim of the present study was the systematic, concentration-dependent evaluation of NO-mediated effects on human HSPC behavior *in vitro*. By culture in the presence of the long-term NO donor diethylenetriamine/nitric oxide adduct (DETA/NO) in a nontoxic concentration window, a biphasic role of NO in the regulation of HSPC behavior was identified: Low DETA/NO concentrations activated classical NO signaling, identified via increased intracellular cyclic guanosine monophosphate (cGMP) levels and proteinkinases G (PKG)-dependent vasodilator-stimulated phosphoprotein (VASP) phosphorylation and mediated a pro-proliferative response of HSPCs. In contrast, elevated NO concentrations slowed cell proliferation and induced HSPC differentiation. At high concentrations, s-nitrosylation levels were elevated, and myeloid differentiation was increased at the expense of lymphoid progenitors. Together, these findings hint at a central role of NO in regulating human HSPC behavior and stress the importance and the potential of the use of adequate NO concentrations for *in vitro* cultures of HSPCs, with possible implications for clinical application of *in vitro* expanded or differentiated HSPCs for cellular therapies.

## Introduction

Tight regulation of proliferation and differentiation is central to blood homeostasis, as its balance secures the life-long regeneration of the blood system. All types of mature blood and immune cells arise from one type of multipotent stem cell termed hematopoietic stem cell (HSC) (Rieger and Schroeder, 2012). By asymmetric cell divisions, HSCs self-renew and generate multipotent progenitors (MPPs) that undergo differentiation and proliferation events to produce all types of mature blood cells but lack the ability to self-renew indefinitely (Beckmann et al., 2007). Additionally, HSCs divide symmetrically to expand the HSC population and ensure the long-term renewal of the blood system (Seita and Weissman, 2010). With ongoing differentiation, hematopoietic stem and progenitor cells (HSPCs) lose their multipotency and are committed to one blood lineage, which are categorized as lymphoid and myeloid (Goardon et al., 2011). Human HSPCs are commonly identified by expression of the surface marker CD34, which is lost on mature blood cells (Civin et al., 1984). By using additional immunophenotypical markers, HSPC subsets can be identified via flow cytometry. As expression of CD38 and CD45RA marks more mature progenitor cells, the lack of these antigens can be used additionally to identify HSCs and MPPs (Lansdorp et al., 1990; Bhatia et al., 1997). During the commitment of lympho-myeloid progenitors to the lymphoid branch, progenitor cells gradually lose myeloid potential with increasing expression of the early B cell marker CD10 (Ichii et al., 2010). Myeloid progenitors (CD34^+^ CD38^+^ CD10^−^) can be grouped into common myeloid progenitors (CMPs), granulocyte-macrophage progenitors (GMPs), and megakaryocyte-erythrocyte progenitors (MEPs) by their expression of CD135 and CD45RA (Doulatov et al., 2010). MEPs lack the expression of CD135, while CMPs and GMPs are both positive for this marker. The transition from CMP to GMP stage is correlated with the expression of CD45RA.

In adult life, HSC behavior is regulated in the red bone marrow by a multitude of signals from neighboring cells, extracellular matrix components, as well as soluble molecules in a highly specialized microenvironment called HSC niche (Asada et al., 2017; Crane et al., 2017; Szade et al., 2018). HSCs are often found in close proximity of the vasculature (Kiel et al., 2005), where distinct types of blood vessels form specialized niche areas (Itkin et al., 2016). A magnitude of signaling molecules has been identified in the niche, which regulate HSC fate. Among these, redox species such as reactive oxygen species (ROS) and nitric oxide (NO) have gained more and more attention (Urao and Ushio-Fukai, 2013; Ludin et al., 2014; Itkin et al., 2016; Ramasamy et al., 2016; Passaro et al., 2017).

NO is a short-lived signaling molecule and is predominantly produced enzymatically *in vivo* by NO synthases (NOS1, NOS2, and NOS3) requiring L-arginine as substrate and O_2_ and reduced nicotinamide adenine dinucleotide phosphate (NADPH) as cosubstrates. The gasotransmitter NO passes cellular membranes by free diffusion, interacts with various intracellular targets depending on its concentration, and thus activates different signaling pathways. At physiological concentrations of NO in the picomolar to nanomolar range (Hall and Garthwaite, 2009), NO primarily interacts with the soluble guanylyl cyclase (sGC) and activates classical NO signaling by binding to its heme center. Subsequently, sGC produces large amounts of cyclic guanosine monophosphate (cGMP), which targets cGMP-dependent proteinkinases G (PKG) among others (Ahern et al., 2002). These serine (Ser)/threonine (Thr) kinases in turn phosphorylate a variety of targets downstream of NO such as the vasodilator-stimulated phosphoprotein (VASP). This actin-binding focal adhesion adaptor protein is implicated in actin reorganization and is predominantly phosphorylated at Ser239 following PKG activation (Thomson et al., 2011). At slightly higher concentrations, NO drives specific posttranslational modifications, termed s-nitrosylation, via the enzyme-independent addition of a nitroso-group to a protein thiol (Martinez-Ruiz et al., 2011). This reversible, redox-sensitive modification has been shown to be implicated in a wide range of physiological processes, while excessive s-nitrosylation is thought to mark pathophysiological modifications. At elevated concentrations NO reacts with other reactive species such as H_2_O_2_ or ${\text{O}}_{2}^{-}$ forming reactive nitrogen species (RNS), which mediate indirect effects of NO. These are thought to account for toxic effects of NO and lead to the induction of apoptosis (Thomas et al., 2008). Due to the short lifetime of the molecule, NO is a locally acting signaling molecule. While it diffuses away from its source, its concentration decreases rapidly as it is quickly oxidized to nitrate and nitrite in aqueous environment.

NO has been identified as a regulator of cell proliferation and differentiation in a variety of cell types (Lv et al., 2015; Tapia-Limonchi et al., 2016) and during organ formation in model organisms (Enikolopov et al., 1999; Peunova et al., 2007). Its impact on HSPC proliferation and differentiation, however, is largely obscure due to contradictory findings in the literature. This originates from a low comparability of the data due to different approaches of NO generation or inhibition of NO signaling. Further, examined species, analyzed cell types, and studied concentration ranges of NO largely differ and thus strongly compromise the ability to draw definite conclusions. To complicate matters further, *in vivo* data generated in mice do not represent the human system accurately, as NO generation differs in rodents and humans, e.g., with regard to the inducibility of NOS2 by inflammatory cytokines and likely with respect to produced NO concentrations (Schneemann and Schoeden, 2007). Moreover, studies based on the pharmacological inhibition of NO synthesis or the *knockout* of NOS isoforms inherently lack the opportunity to monitor concentration-dependent effects of NO. This is crucial, however, as NO signaling is highly concentration dependent. As the HSC niche is comprised of a complex communication network where various cell types could act as NO sources and NO could interact with a multitude of targets to yield indirect effects, direct effects of NO on human HSPC proliferation and differentiation were monitored *in vitro*. By systematic variation in NO levels in a nontoxic concentration range, NO signaling was mimicked in various settings such as homeostasis and acute stress. As mode of NO generation the long-term donor diethylenetriamine/nitric oxide adduct (DETA/NO)—a diazeniumdiolate—was chosen, which spontaneously releases 2 mol NO per mol of the parent compound and has a half-life of 20 h under physiological conditions. This is described to yield a concentration of free NO, which is estimated to be three orders of magnitude lower than the parent compound (Pervin et al., 2007). By covering a wide concentration range of physiologically relevant concentrations of NO, this study aims to shed light onto the concentration-dependent effects of NO signaling in human HSPCs *in vitro* and to contribute to a better understanding of NO-mediated effects under homeostasis and acute stress in the hematopoietic system.

## Materials and Methods

### Chemicals

All chemicals, unless otherwise stated, were obtained from Merck KGaA, Darmstadt, Germany and used as obtained.

### Isolation and *in vitro* Culture of Human CD34^+^ Cells

Human umbilical cord blood was obtained from DKMS cord blood bank gGmbH (Dresden, Germany) or the cord blood bank of the German Red Cross Services (Mannheim, Germany) after informed consent of the parents and approval by the local ethics committee (Ethik-Kommission der Landesärztekammer Baden-Württemberg, project number B-F-2013-111). Mononuclear cells were isolated from cord blood samples up to 48 h after collection by density gradient centrifugation using lymphocyte separation medium (PromoCell GmbH, Heidelberg, Germany). Subsequently, HSPCs were obtained from the mononuclear cell fraction by positive selection for the cell surface marker CD34 via magnetic activated cell sorting (MACS, Miltenyi Biotec GmbH, Bergisch Gladbach, Germany) according to the manufacturer's instructions. HSPCs were used for experiments if the CD34^+^ cell fraction was at least 95% as determined by flow cytometry. HSPCs were maintained in Hematopoietic Progenitor Expansion Medium DXF (PromoCell GmbH, Heidelberg, Germany) with 1% of Cytokine Mix E (PromoCell GmbH, Heidelberg, Germany) in a humidified incubator at 37°C and 5% CO_2_.

### Stimulation of HSPCs With DETA/NO

DETA/NO stock solutions were made in 0.01 M NaOH, and the optical density (OD) of the intact NO donor was checked at λ = 252 nm via UV/vis (ε = 7,640 M^−1^ cm^−1^). Aliquoted stock solutions were kept at −80°C for several days. Before use in experiments, the concentration of the stock solution was assessed via UV/vis and further diluted in cool phosphate-buffered saline (PBS) to 1,000 × of the intended concentrations. Cells were stimulated with 1 μl/ml in cell culture medium. To generate a prolonged release of NO and avoid accumulation of nitrite and nitrate as by-products of NO release, a media exchange was performed daily with stimulation.

### Flow Cytometric Analysis of HSPC Proliferation and Differentiation

HSPCs were stained with 1 μM CellTrace™ Violet (CTV, Thermo Fisher Scientific Inc. Waltham, MA, USA) according to the manufacturer's instructions after isolation. HSPCs were stimulated daily with up to 20 μM of DETA/NO. On days 0 and 5, cells were additionally stained for the expression of cell surface markers using fluorescently labeled antibodies and analyzed by flow cytometry.

For analysis of cell surface markers 4 × 10^4^ CTV-stained HSPCs were seeded in 1 ml Hematopoietic Progenitor Expansion Medium DXF (PromoCell GmbH) with 1% of Cytokine Mix E (PromoCell GmbH) in a 24-well plate and stimulated daily with up to 20 μM of DETA/NO. On days 0 and 5 of culture, HSPCs were additionally stained with the following antibody cocktail (*clone*; dilution): anti-human CD34-PE-Cy7 (*581*; 1:40), anti-human CD38-APC-H7 (*HB7*; 1:20), anti-human CD10-APC (*HI10a*; 1:20), anti-human CD45RA-FITC (*HI100*; 1:5), and anti-human CD135-PE (*4G8*, 1:5) (all antibodies from Becton Dickinson Inc., East Rutherford, NJ, USA) according to manufacturer's instructions. 7AAD (1:80; Becton Dickinson Inc.) was added for the last 10 min of antibody incubation. For discrimination of positive populations, 1% of fluorescence-minus-one controls (FMO controls) were regarded as false positive events. Cells were analyzed using the BD FACSVerse^TM^ flow cytometer with 405, 488, and 640 nm lasers at a flowrate of 60 μl min^−1^. Photomultiplier voltages were adjusted using unstained cells, and fluorescence spillover from one channel into another was compensated using single-stained controls. For the analysis of HSPC proliferation and differentiation, FlowJo 7.6.5 (FlowJo LLC, Ashland, OR, USA) was used. Singlet events were discriminated from aggregates using the pulse-width against area of the forward scatter (FSC) signal. HSPCs were gated in a dot plot of the side scatter against the FSC. Proliferation data were obtained via the CTV signal using the *proliferation tool* algorithm in FlowJo, where the CTV signal on day 0 was fixed as peak 0 and the number of generations as well as the variation coefficient was kept constant for all samples. Cellular events in each cell generation were used to calculate the fraction of cells in each cell generation as well as the cell division index. The latter is defined as the number of cellular divisions divided by the initial cell number. For the analysis of HSPC differentiation, viable CD34^+^ cells (7AAD^−^, CD34^+^) were categorized into HSCs/MPPs (7AAD^−^, CD34^+^; CD38^−^, CD45RA^−^), CLPs (7AAD^−^, CD34^+^; CD10^+^) and myeloid progenitors (7AAD^−^, CD34^+^; CD10^−^; CD38^+^) according to their surface marker expression via FMO controls. Accordingly, myeloid progenitors were further divided into CMPs (CD135^+^, CD45RA^−^), GMP (CD135^+^, CD45RA^+^), and MEPs (CD135^−^, CD45RA^−^) depending on their expression of CD135 and CD45RA. Cell fractions in single HSPC populations were exported to GraphPad Prism 6 (GraphPad Software, San Diego, CA, USA) for further analysis. Due to high interdonor variations, data were additionally normalized to the solvent control for better comparability. Mean values of *n* = 4 independent experiments as well as the corresponding standard deviations were calculated using GraphPad Prism and analyzed for significance of intermean differences via analysis of variance (ANOVA).

### Colony Forming Unit Assay

Myeloid progenitor cells were determined retrospectively by a colony forming unit assay. HSPCs (4 × 10^4^) were seeded in 1 ml medium in a 24-well plate and stimulated daily with up to 20 μM DETA/NO for 5 days with daily media exchange. On days 0 and 5 of culture, 1,500 cells were transferred to 300 μl of Iscove's modified Dulbecco's medium with 2% fetal bovine serum (Stemcell Technologies Inc., Vancouver, Canada), mixed thoroughly with 3 ml of MethoCult H4434 (Stemcell Technologies Inc.), and plated into 35 mm Petri dishes in triplicates, which were kept under standard cell culture conditions for 12 days. The colonies were assessed by light microscopy (Axio Vert.A1, Carl Zeiss AG, Oberkochen, Germany) and characterized according to the “Atlas of Human Hematopoietic Colonies From Cord Blood” (Stemcell Technologies Inc., Vancouver, Canada).

### Competitive Enzyme-Linked Immunoassay to Determine Intracellular cGMP Levels

Intracellular cGMP levels of HSPCs after stimulation with DETA/NO were determined using the Cyclic GMP XP® Assay Kit (Cell Signaling Technology Inc., Danvers, MA, USA) following manufacturer's instructions. After cultivation at a density of 10^5^ cells × ml^−1^ for 24 h, 10^5^ cells were treated with up to 25 μM of DETA/NO alone or additionally pretreated with 25 molar equivalents of 2-4-carboxyphenyl-4,4,5,5-tetramethylimidazoline-1-oxyl-3-oxide (cPTIO) for 10 min (compared to the maximal amount of NO released by DETA/NO). Cells were incubated for 30 min at 37°C and 5% CO_2_ after treatment and lysed following the manufacturer's instructions using the supplied lysis buffer supplemented with 1 mM phenylmethylsulfonyl fluoride and stored at −20°C. The assay was performed with the Cyclic GMP XP® Assay Kit (Cell Signaling Technology Inc., Danvers, MA, USA) using 40 μl of the lysates or standard together with 40 μl of the horseradish peroxidase (HRP)-coupled cGMP. For data analysis, the measured optical density (OD) values at λ = 450 nm, which correspond to the converted substrate by the HRP-coupled competitive antigen, were exported to a spreadsheet program and used for data analysis.

### Detection of Protein Phosphorylation via Western Blotting

After cultivation at a density of 10^5^ cells × ml^−1^ for 24 h, 10^5^ cells were treated with up to 25 μM DETA/NO directly or stimulated with 10 μM DETA/NO and preincubated with 25 molar equivalents of cPTIO for 10 min (respective to the maximal amount of NO released by DETA/NO). After 30 min of stimulation, cells were lysed with lysis buffer [20 mM Tris–HCl, 150 mM NaCl, 1 mM ethylenediaminetetraacetic acid (EDTA), 1 mM ethylene glycol tetraacetic acid (EGTA), 1% Triton X-100] on ice for 15 min. Equal protein amounts were mixed with Laemmli buffer, heated (95°C, 5 min), loaded onto 12% acrylamide gels, separated by size via sodium dodecyl sulfate polyacrylamide gel electrophoresis (SDS-PAGE) and blotted onto polyvinylidene fluoride (PVDF) membranes (Bio-Rad Laboratories Inc., Hercules, USA) via wet blotting. Membranes were blocked with 5% bovine serum albumin (BSA) in Tris-buffered saline (20 mM Tris–HCl, 140 mM NaCl, pH = 7.6) with 0.1% Tween 20 (TBST) (1 h, RT) and incubated with rabbit anti-human VASP phosphoSer239 (polyclonal, 1:2,000; Cell Signaling Technology Inc., Danvers, MA, USA), mouse anti-human VASP (*43/VASP*, 1:1,000; Becton Dickinson Inc., East Rutherford, NJ, USA) or mouse anti-human Vinculin (*hVIN-1*, 1:400; Merck KGaA, Darmstadt, Germany) at 4°C overnight. After extensive washing in TBST, the membranes were incubated with species-specific, HRP-conjugated secondary antibodies (1:20,000; Cell Signaling Technology Inc., Danvers, MA, USA) (1 h, RT). Subsequently membranes were washed thoroughly in TBST, incubated with the SuperSignal™ West Femto Maximum Sensitivity substrate (Thermo Fisher Scientific Inc., Waltham, MA, USA) according to the manufacturer's instructions, and the chemiluminescence signal was detected using a Lumi-Imager™ F1 detection system (Roche Diagnostics International AG, Risch, Switzerland). After linear adjustment of brightness and contrast, grayscale values of the protein bands were determined in the regions of interest using the *gel analysis* tools in ImageJ (https://imagej.nih.gov/ij/index.html). To determine the signal independent of the protein amount, gray values of the protein bands of interest were normalized to the respective loading controls.

### Detection of Protein s-Nitrosylation via Western Blotting

Protein s-nitrosylation of HSPCs after 5 days of stimulation with up to 20 μM of DETA/NO was assessed with the Pierce™ S-Nitrosylation Western Blot Kit (Thermo Fisher Scientific Inc., Waltham, MA, USA) according to the manufacturer's instructions with protein precipitation steps performed overnight. Chemiluminescence signals of the membranes were detected using a Lumi-Imager™ F1 detection system (Roche Diagnostics International AG, Risch, Switzerland). After linear adjustment of brightness and contrast, grayscale values of the protein bands were determined in the regions of interest using the *gel analysis* tools in ImageJ (https://imagej.nih.gov/ij/index.html). To determine the signal independent of the protein amount, gray values of the protein bands of interest were normalized to the respective loading controls.

### Expression of Nitric Oxide Synthases via Polymerase Chain Reaction

RNA was isolated from the positive control cell lines HaCat (obtained by the German Cancer Research Center), CaCo2 (kind gift by Prof. Orian-Rousseau, Karlsruhe Institute of Technology), and THP-1 (kind gift by Prof. Overhage, former Karlsruhe Institute of Technology, now: Carlton University, Canada), the HSPC model cell lines KG1a and TF-1 (both Leibniz-Institut-Deutsche Sammlung von Mikroorganismen und Zellkulturen GmbH), freshly isolated human HSPCs derived from cord blood of five donors, as well as mesenchymal stromal cells (MSCs) derived from bone marrow (kind gift by Prof. Bieback, Medical Faculty Mannheim, University Heidelberg) of three donors using the RNeasy® Mini Kit (Quiagen N.V., Venlo, Netherlands) according to the manufacturer's instructions. RNA concentration was determined by measuring the optical density of the obtained samples at λ = 280 nm via a NanoDrop™ 2000 spectrophotometer (Thermo Fisher Scientific Inc., Waltham, MA, USA). Complementary DNA (cDNA) was synthesized from the isolated RNA samples applying the TaqMan™ Reverse Transcription Reagents (Thermo Fisher Scientific Inc., Waltham, MA, USA). Via PCR, specific gene sections were amplified using the peqGOLD Hot Taq-DNA Polymerase Kit and dNTP Set (both VWR International GmbH, Darmstadt, Germany) as well as specific primers for NOS1 (forward, CCTCCCGCCCTGCACCATCTT; reverse, CTTGCCCCATTTCCATTCCTCGTA), NOS2 (forward, TCCGAGGCAAACAGCACATTCA; reverse, GGGTTGGGGGTGTGGTGATGT), and NOS3 (forward, CATCTTCAGCCCCAAACGGA; reverse, AGCGGATTGTAGCCTGGAAC). As a negative control, a no template control with ultrapure RNase-free water instead of template was carried along. After heating to 95°C for 30 s, DNA sections were amplified in 30 cycles of denaturation (94°C; 30 s), annealing (NOS1/NOS3, 60°C; NOS2, 62°C; 30 s), and elongation (72°C, 90 s) and subsequently cooled to 4°C. DNA products were mixed with DNA Gel loading dye (6x, Thermo Fisher Scientific Inc., Waltham, MA, USA) and separated via agarose gel electrophoresis in a 2% (w/v) agarose gel with GelRed™ Nucleic Acid Gel Stain (Biotium, Inc., Hayward, CA, USA). For size determination of the fragments, the DNA ladder 100 bp as well as 1 kb plus (VWR International GmbH, Darmstadt, Germany) was applied. The gels were analyzed using the Lumi-Imager™ F1 detection system (Roche Diagnostics International AG, Risch, Switzerland).

### Flow Cytometric Analysis of Apoptotic and Dead Cells via Annexin V and Sytox™ AADvanced™ (Sytox) Staining to Determine a Cytocompatible Concentration Range of DETA/NO

On the day of isolation, 2 × 10^4^ HSPCs were seeded in 500 μl of cell-specific culture medium in a 48-well plate and stimulated with up to 500 μM DETA/NO. Cells were cultured for 5 days with daily media renewal and stimulation with the NO donor. On day 5, the cell number was determined and 2–10 × 10^4^ cells were washed in annexin buffer (10 mM HEPES, 140 mM NaCl, 2.5 mM CaCl_2_, pH = 7.4). After centrifugation, the cell pellet was resuspended in 100 μl annexin buffer with 1 μM Sytox™ AADvanced™ (Sytox, Thermo Fisher Scientific Inc., Waltham, MA, USA) and 5 μl Annexin V-FITC (BioLegend Inc., San Diego, CA, USA) and incubated for 15 min at RT protected from light. After washing with 500 μl annexin buffer, cells were immediately analyzed by flow cytometry. Single stain controls, with heat-treated and untreated cells, were used for compensation. The viable cell fraction (Annexin V^−^ and Sytox^−^) was plotted against the decadic logarithm of the DETA/NO concentration, and the data points were interpolated using the hill function below to determine the mean effective concentration EC_50_.

$$\begin{array}{l} y=\;\frac{\left(A_{1}-A_{2}\right)}{\left\{1+10^{\left[\log\left(EC_{50}\right)-x\right]}\right\}} \end{array}$$

### Statistics

Statistical significance of intermean differences was tested with one-way ANOVA using GraphPad Prism 6 (GraphPad Software, San Diego, CA, USA). Tests were chosen according to the requirements of the respective experiment and data sets. Paired data sets were subjected to one-way ANOVA with Greenhouse–Geisser correction. When comparing multiple intermean differences in comparison to a control group, Dunnett's test was applied to correct for multiple comparisons. Additionally, a posttest was applied to test for linearity of the group means with increasing concentration of the added compound. When comparing preselected pairs of columns, Sidak's test was used to correct for multiple comparisons. *P* < 0.05 were regarded as indicative for statistically significant differences between compared groups and are marked by an asterisk (^*^) in the respective figures, *P* < 0.01 or 0.001 by two asterisks (^**^) or three asterisks (^***^), respectively, and *P* > 0.05 by “ns” (not significant).

## Results

### NO Release Influences the Proliferation of HSPCs in a Concentration-Dependent Manner

**Figure 1:**
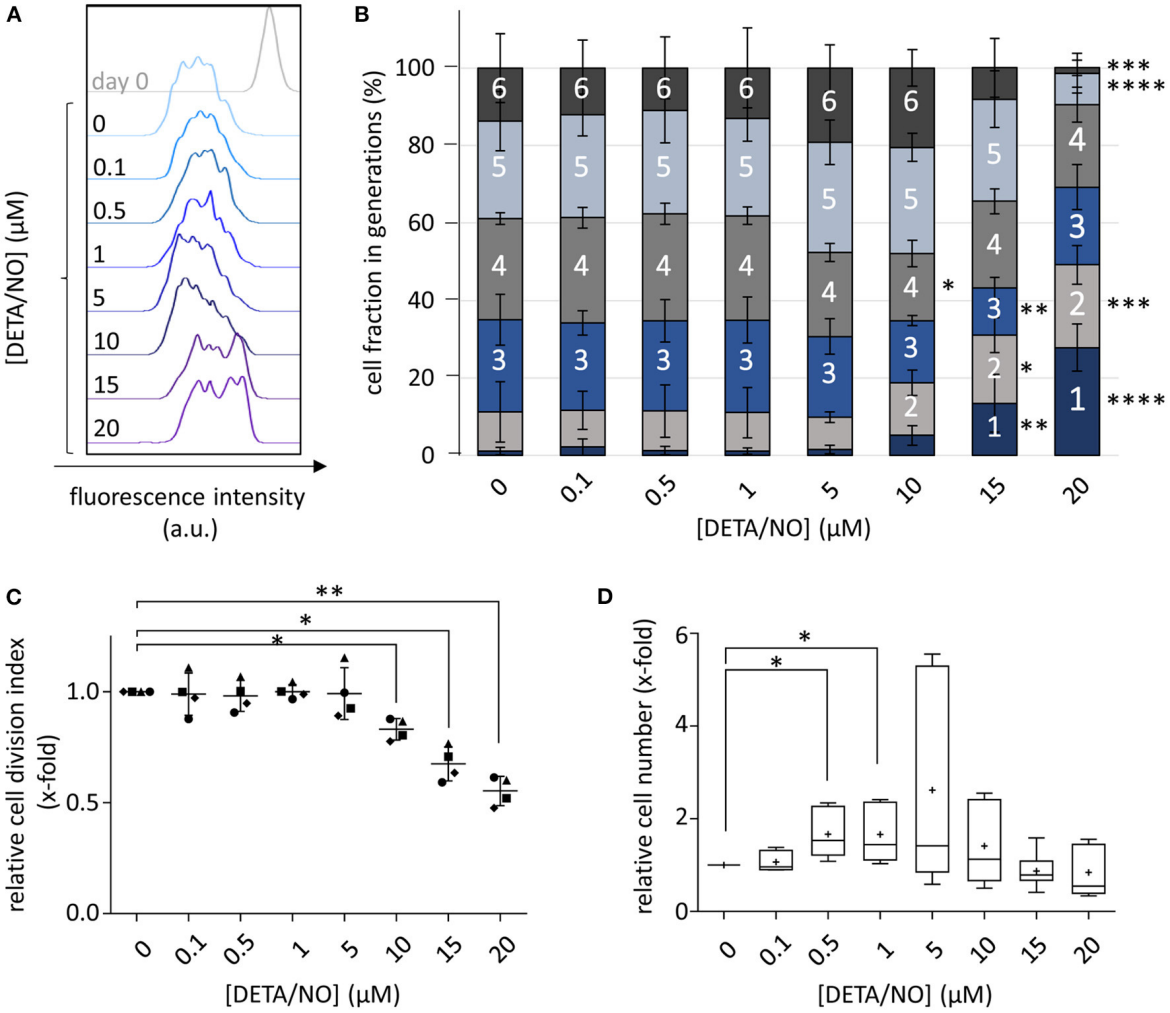
Concentration-dependent influence of up to 20 μM diethylenetriamine/nitric oxide adduct (DETA/NO) on hematopoietic stem and progenitor cell (HSPC) proliferation *in vitro*. **(A)** Overlay of the CellTrace^TM^ Violet (CTV) fluorescence intensity profiles (x-axis) of one representative donor before (gray, top) and after cultivation with DETA/NO for 5 days (colored, concentrations increasing from top to bottom). **(B)** Calculated cell fractions in HSPC generations (y-axis) after 5 days of culture with DETA/NO (x-axis). Bar graphs indicate the mean cell fraction in single cell generations (indicated by numbers) of *n* = 4 independent experiments with the corresponding standard deviation as error bars. **(C)** Calculated cell division index of CTV-stained HSPCs relative to the solvent control (y-axis) after 5 days of stimulation with DETA/NO (x-axis). The dot plot displays the relative cell division index of *n* = 4 independent experiments with the mean and the corresponding standard deviation shown as line and error bars. **(D)** Cell number of unstained HSPCs normalized to the solvent control (y-axis) after 5 days of culture in the presence of DETA/NO (x-axis). The boxplot graph displays the median as a line across the boxes of *n* = 8 independent experiments and the mean as a plus. Lower and upper boxes indicate the 25th percentile to the 75th percentile. Whiskers represent maximum and minimum values. Statistically significant intermean differences as per ANOVA (comparison of **(A–C)** each column with the control or **(D)** preselected pairs of columns) are indicated as follows: *P < 0.05; **P < 0.001; ***0.0001 < *P* < 0.001; ****P < 0.0001.

To systematically monitor the concentration-dependent influence of NO on HSPC proliferation, cells were stained with CellTrace™ Violet (CTV) after isolation, cultured in the presence of DETA/NO for 5 days and subsequently analyzed via flow cytometry. As concentrations up to 20 μM of DETA/NO were well tolerated by HSPCs (Supplementary Figure 1), they were used for cell stimulation to cover a wide concentration range of the gasotransmitter. Additional inhibition of inherent NO synthesis with small molecules was not required in the present study, as human cord blood-derived HSPCs did not express NO synthase isoforms (Supplementary Figure 2). Stimulation with various concentrations of DETA/NO affected the proliferative activity of HSPCs in a biphasic manner. As shown in Figure 1A for a representative donor, the CTV signal was shifted to lower fluorescence intensities at intermediate DETA/NO concentrations (1 and 5 μM) in comparison to the solvent control, revealing a higher proliferative activity of HSPCs during culture. In contrast, concentrations above 10 μM DETA/NO caused a decreased proliferative activity that lead to a retention of CTV. Further, at elevated concentrations of DETA/NO, the emergence of two HSPC subpopulations of different proliferative activity was observed. This also manifested itself in the HSPC distribution in the respective cell generations. Culture with low DETA/NO concentrations had no significant impact on the distribution of mean cell fractions, while elevated concentrations starting at 10 μM DETA/NO induced a dramatic change (Figure 1B). At these levels, cell fractions that underwent one to two cell divisions were increased at the expense of highly proliferative cells. In addition, HSPCs that had performed three or four cell divisions were reduced when treated with 10 or 15 μM DETA/NO, respectively. At 20 μM DETA/NO, this increase was at the expense of highly proliferative HSPC fractions, which underwent more than four cell divisions. This effect was also prominent in the calculated cell division index. Relative to the solvent control, the index was significantly reduced to 0.83-fold upon culture in presence of 10 μM DETA/NO (Figure 1C). Higher DETA/NO levels induced an even stronger decrease (0.55-fold of the control at 20 μM DETA/NO). These findings were complemented by analysis of the NO-induced proliferative behavior of unstained HSPCs (Figure 1D). Here, culture conditions of 0.5 and 1 μM DETA/NO lead to a significant increase in cell number to the 1.67- and 1.66-fold relative to the solvent control after 5 days of culture, while high concentrations of DETA/NO seemed to have a reductive effect on the cell number. Together, these results indicate a concentration-dependent effect of NO on human HSPC proliferation *in vitro*. Despite the lack of a significant, NO-induced change in cell proliferation at intermediate DETA/NO levels, the NO-induced increase in HSPC numbers adds to the observed pro-proliferative tendencies detected via CTV staining at these NO levels. Thus, these data support a biphasic role of NO on HSPC proliferation where low to intermediate NO concentrations encourage HSPC proliferation while higher levels slow cell proliferation.

### Prolonged NO Release Increases the Expression Level of CD34 on HSPCs

**Figure 2:**
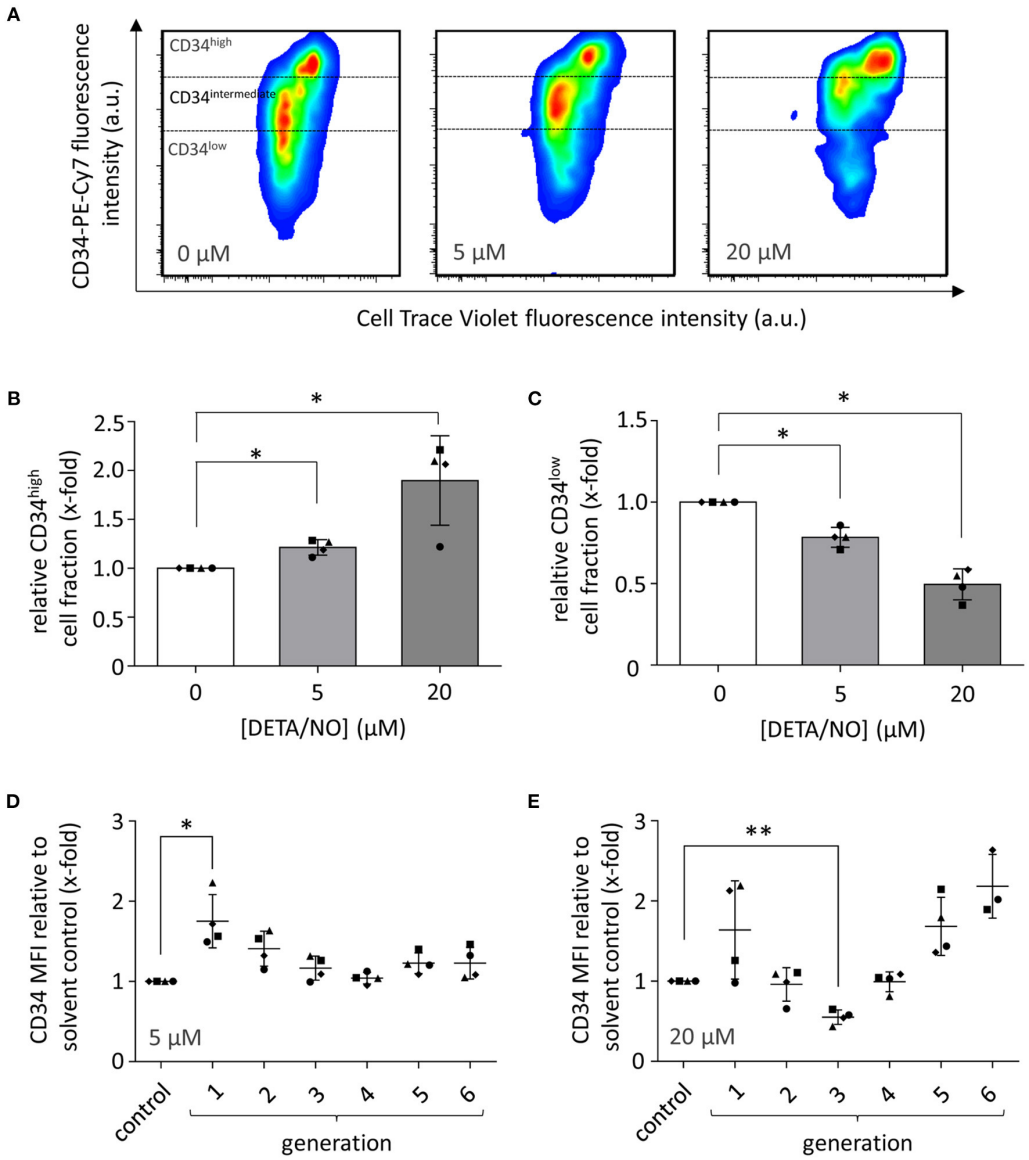
Effect of hematopoietic stem and progenitor cell (HSPC) culture in presence of diethylenetriamine/nitric oxide adduct (DETA/NO) for 5 days on the expression of the HSPC marker CD34 as analyzed by flow cytometry. **(A)** Pseudo-colored, smoothed dot plots of the fluorescence intensity in the CD34 channel (y-axis) shown against the CellTrace^TM^ Violet (CTV) channel (x-axis) for one representative donor after culture in presence of 0, 5, and 20 μM of DETA/NO. Expression levels of CD34 were categorized in CD34^high^, CD34^intermediate^, and CD34^low^ depending on the displayed fluorescence intensity as indicated. **(B)** CD34^high^ and **(C)** CD34^low^ cell fractions (y-axis) after culture with 0, 5, and 20 μM of DETA/NO (x-axis). Bar graphs display the mean cell fractions of *n* = 4 independent experiments with the corresponding standard deviations as error bars. Effect of stimulation with **(D)** 5 μM and **(E)** 20 μM DETA/NO on the expression level of CD34 in cells of different proliferative activity. The y-axis displays the mean fluorescence intensity (MFI) in the CD34 channel relative to the solvent control, while on the x-axis, the solvent control (control) as well as the number of cellular divisions are shown indicated as generations. Dot plots display the mean of *n* = 4 independent experiments with corresponding standard deviations as lines with error bars. Statistically significant intermean differences as per ANOVA (comparison of each column with the control) are indicated as follows: *P < 0.05; **P < 0.001.

We next asked which cell types and processes within the applied HSPC populations were responsible for the observed changes in HSPC proliferation: rather HSCs and MPPs indicating that their maintenance was induced by NO or rather early progenitor cells, suggesting that differentiation was enhanced in response to NO. Thus, we additionally monitored the expression of the HSPC marker CD34 after culture with 5 and 20 μM DETA/NO and compared it to the solvent control. The CD34-PE-Cy 7 fluorescence intensity as a measure of CD34 expression was increased by treatment with DETA/NO (Figure 2A). Highly proliferative cell fractions that display low to intermediate CD34 fluorescence intensity when treated with the solvent alone were shifted to higher CD34 fluorescence intensities with DETA/NO treatment both at intermediate and high concentrations. To better quantify this effect, we categorized HSPCs according to their CD34 expression in CD34^high^, CD34^intemediate^, and CD34^low^ based on the fluorescence intensity in the respective channel (Figure 2B). CD34^high^ cell fractions were increased significantly by 1.2- and 1.9-fold compared to the solvent control after culture with 5 and 20 μM DETA/NO, respectively. Accordingly, DETA/NO treatment decreased CD34^low^ cell fractions to 0.8- and 0.5-fold of the solvent control at intermediate and high concentrations (Figure 2C). The increase in CD34 expression was not distributed equally over the total HSPC population but differed depending on the proliferative state of the cells (Figures 2D,E). Compared to the solvent control, intermediate DETA/NO concentrations induced a significant increase in CD34-PE-Cy 7 mean fluorescence intensity (MFI) in cell fractions, which underwent low numbers of cellular divisions. While at high DETA/NO concentrations, this trend was also observable, the CD34 MFI increased additionally with increasing proliferative activity, while cells that underwent three cellular divisions showed a significant decrease in CD34 MFI to 0.6-fold compared to the solvent control. This emergence of two highly CD34 positive subpopulations of different proliferative state hinted at a NO-induced differentiation of HSPCs rather than HSC expansion. This hypothesis was also supported by the observation that both the CD34^+^ cell fraction and total CD34 expression levels were reduced after less cell generations when compared to the control (Supplementary Figure 3). Based on these observations, we concluded that the concentration-dependent increase in CD34^high^ expression by DETA/NO could not have been caused solely by an expansion of HSCs, as this would have caused a retention of CD34 expression for more cell generations in comparison to the control. Instead, the data showed the contrary behavior and additionally hinted toward the generation of two subpopulations, which strongly express CD34 and proliferate at different rates. We thus hypothesized that NO affects the differentiation behavior of HSPCs in the culture in a concentration-dependent manner. To test this, we employed a multicolor flow cytometry panel and analyzed the different HSPC subpopulations according to their characteristic antigen expression profile.

### NO Supports Differentiation Into Myeloid Progenitors *in vitro*

**Figure 3:**
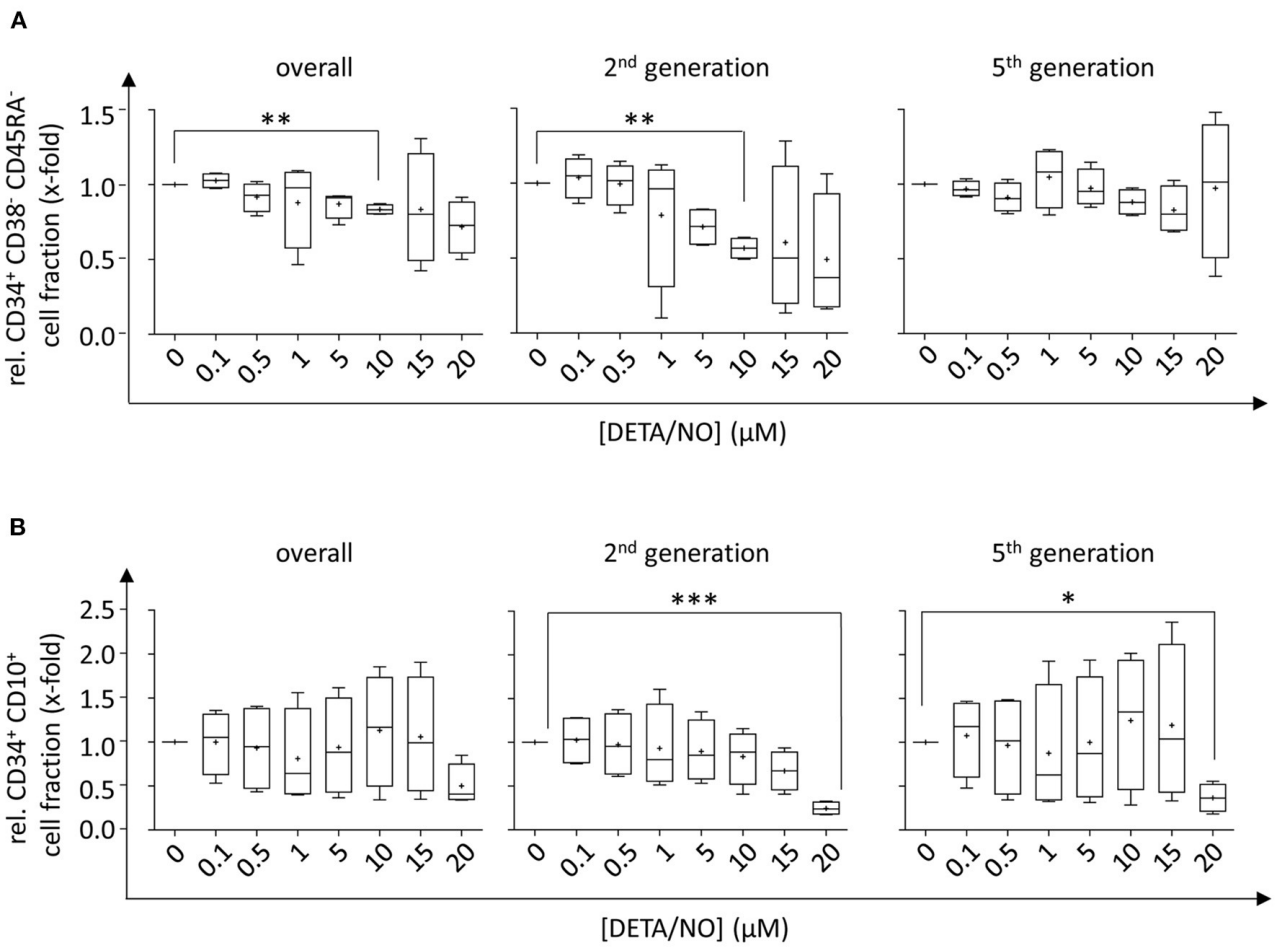
Influence of up to 20 μM diethylenetriamine/nitric oxide adduct (DETA/NO) on the retention of hematopoietic stem cell (HSC) and multipotent progenitor (MPP) fractions and the differentiation behavior into early lymphoid progenitor populations *in vitro* as determined by flow cytometry. Fractions of **(A)** CD34^+^ CD38^−^ CD45RA^−^ HSC and MPP fractions and **(B)** CD34^+^ CD10^+^ CLP fractions (y-axis) relative to the solvent control after 5 days of culture in presence of DETA/NO in the overall hematopoietic stem and progenitor cell (HSPC) population regardless of their proliferative activity (left) and in cell fractions that underwent two (middle) and five (right) cell divisions. Boxplot graphs display the median as a line across the boxes of *n* = 4 independent experiments with mean values indicated by a plus. Lower and upper boxes indicate the 25th to the 75th percentile. Whiskers represent maximum and minimum values. Statistically significant intermean differences as per ANOVA (comparison of each column with the control) are indicated as follows: *P < 0.05; **P < 0.001; ***0.0001 < *P* < 0.001.

**Figure 4:**
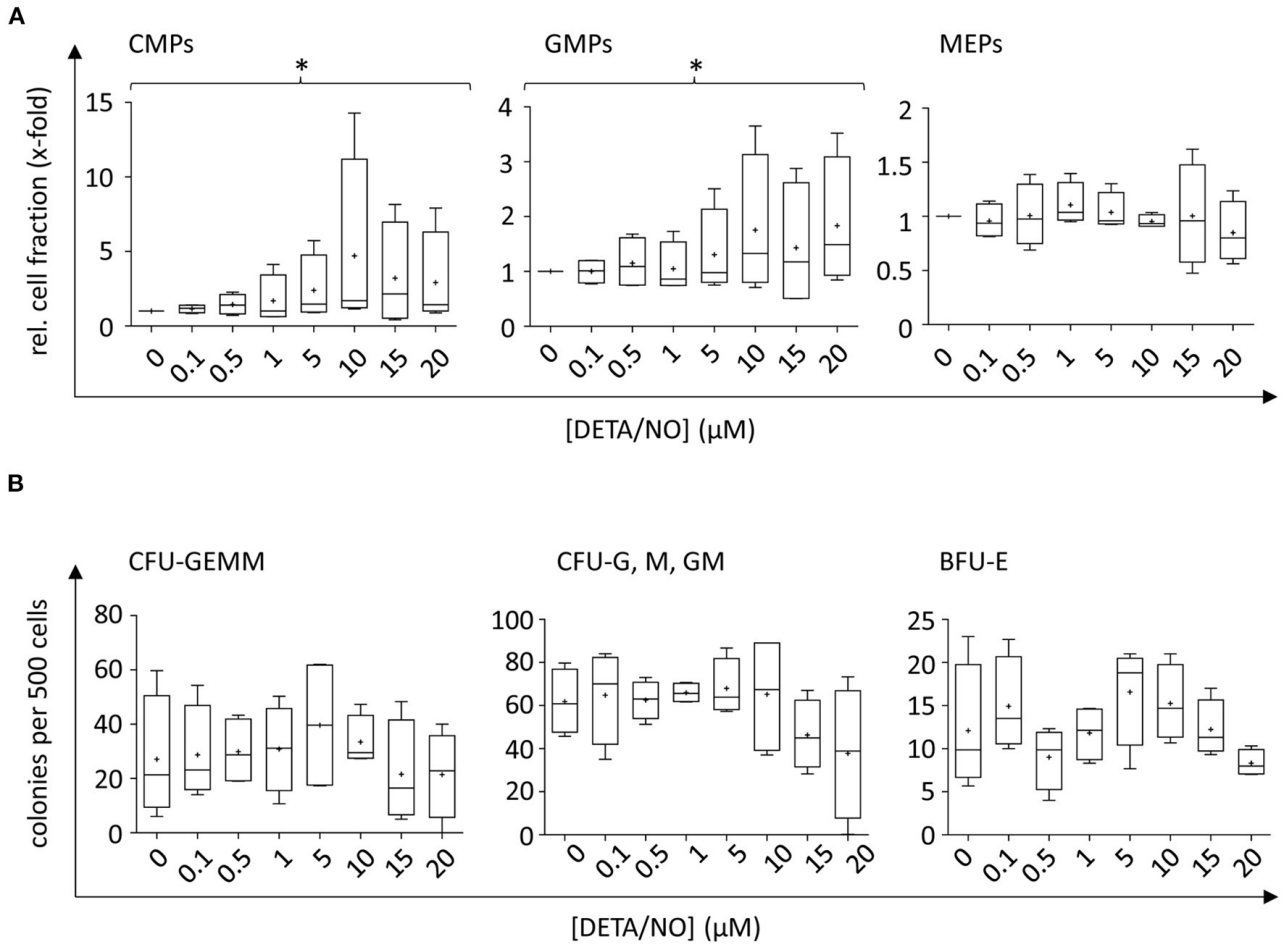
Concentration-dependent effect of up to 20 μM diethylenetriamine/nitric oxide adduct (DETA/NO) on myeloid differentiation of human hematopoietic stem and progenitor cells (HSPCs) *in vitro*. **(A)** Myeloid cell fractions relative to the solvent control (y-axis) as determined immunophenotypically by flow cytometry depending on the used concentration of DETA/NO in the culture (x-axis). Myeloid progenitors were identified in the CD34^+^ CD38^+^ CD10^−^ HSPC subset by their differential expression of CD135 and CD45RA, where CMPs, GMPs, and MEPs were CD135^+^ CD45RA^−^, CD135^+^ CD45RA^+^, and CD135^−^ CD45RA^−^, respectively. **(B)** Myeloid progenitors as determined by a colony-forming unit (CFU) assay. The number of colonies per 500 plated cells (y-axis) is shown dependent on the DETA/NO concentration during culture (x-axis). Boxplot graphs display the median as a line across the boxes of *n* = 4 independent experiments with the mean shown as a plus. Lower and upper boxes indicate the 25th to the 75th percentile. Whiskers represent maximum and minimum values. Statistically significant intermean differences as per ANOVA (posttest for linear trend of the group means) are indicated as follows: *P < 0.05.

To analyze NO-mediated effects on HSPC differentiation, the expression of the cell surface markers CD34, CD38, CD45RA, CD10, and CD135 was assessed in addition to monitoring the proliferative behavior by dye dilution of CTV via flow cytometry (for detailed gating strategy, see Supplementary Figure 4 in the Supplementary Material). HSCs and MPPs were identified from vital CD34^+^ cells using FMO controls as displayed in Figure 3A. The distinction of HSC and MPP populations via analysis of CD90 and CD49f coexpression was omitted in favor of CTV incorporation into the panel due to limited cell numbers and low expression of CD90 and CD49f in CD34^+^ subsets in previous experiments. In the presence of DETA/NO, a concentration-dependent decrease in HSC and MPP fractions was observed independent of their proliferative activity, and a significant reduction to 0.83-fold of the control was induced by stimulation with 10 μM DETA/NO. This was due to a marked reduction in HSCs and MPPs in HSPC subsets with low proliferative activity (second generation), while there was no observable effect in highly proliferative HSPCs (as exemplified by HSPCs after five cell divisions). Reduced numbers of HSCs and MPPs with low proliferative activity could have been caused by an increased proliferation of these early HSPC types; however, this would have resulted in elevated HSC and MPP numbers in cell fractions that underwent higher numbers of cell division. As NO only affected HSC and MPP numbers in cell fractions that cycled at a low rate without having any impact on higher proliferative HSC and MPP fractions, NO seemed to drive HSPCs into differentiation in a concentration-dependent fashion. CD34^+^ CD10^+^ common lymphoid progenitors (CLPs) were reduced by treatment of 20 μM DETA/NO both in weakly and highly proliferative cell subsets (Figure 3B). Here, CLP fractions were reduced to 0.24- and 0.37-fold of the solvent control in HSPC fractions that underwent two and five cell divisions, respectively. Early myeloid progenitor cells were also affected by treatment with DETA/NO. CMP and GMP fractions increased with rising concentration of DETA/NO during culture (Figure 4A). However, MEP cell fractions were not significantly affected by treatment with DETA/NO in the overall cell population. Yet, MEP fractions were significantly increased in weakly cycling HSPCs in a dose-dependent manner, while GMP fractions were reduced (Supplementary Figure 5A). In HSPC fractions of higher proliferative activity, MEP fractions were not affected, while CMP and GMP fractions were increased with increasing concentration of DETA/NO (Supplementary Figure 5B). This influence of NO on early myeloid progenitors was only partially reflected by functional assays. Colony forming unit assays, which enable the retrospective enumeration of myeloid progenitors, revealed no significant influence of DETA/NO stimulation on myeloid colony-forming units (CFUs) due to a high variance of colony numbers (Figure 4B). Yet, myeloid colony numbers seemed to rise to a local maximum at culture with 5 μM DETA/NO, which was seen in CFU-GEMM, CFU-G,M,GM, and BFU-E. At higher concentrations of 15 and 20 μM, myeloid colony numbers were reduced to solvent control levels. Taken together, these observations support the hypothesis that NO affects the differentiation behavior of HSPCs in a concentration-dependent manner. Rather than supporting HSC and MPP expansion NO seems to act as a concentration-dependent driver of differentiation. At the highest tested concentrations of DETA/NO, differentiation into lymphoid progenitors was markedly reduced, while highly proliferative myeloid cell fractions were supported. In functional assays, however, these myeloid progenitors showed a decreased CFU-forming activity.

### NO Activates Predominantly Classical Signaling Pathways at Low and Intermediate Levels

**Figure 5:**
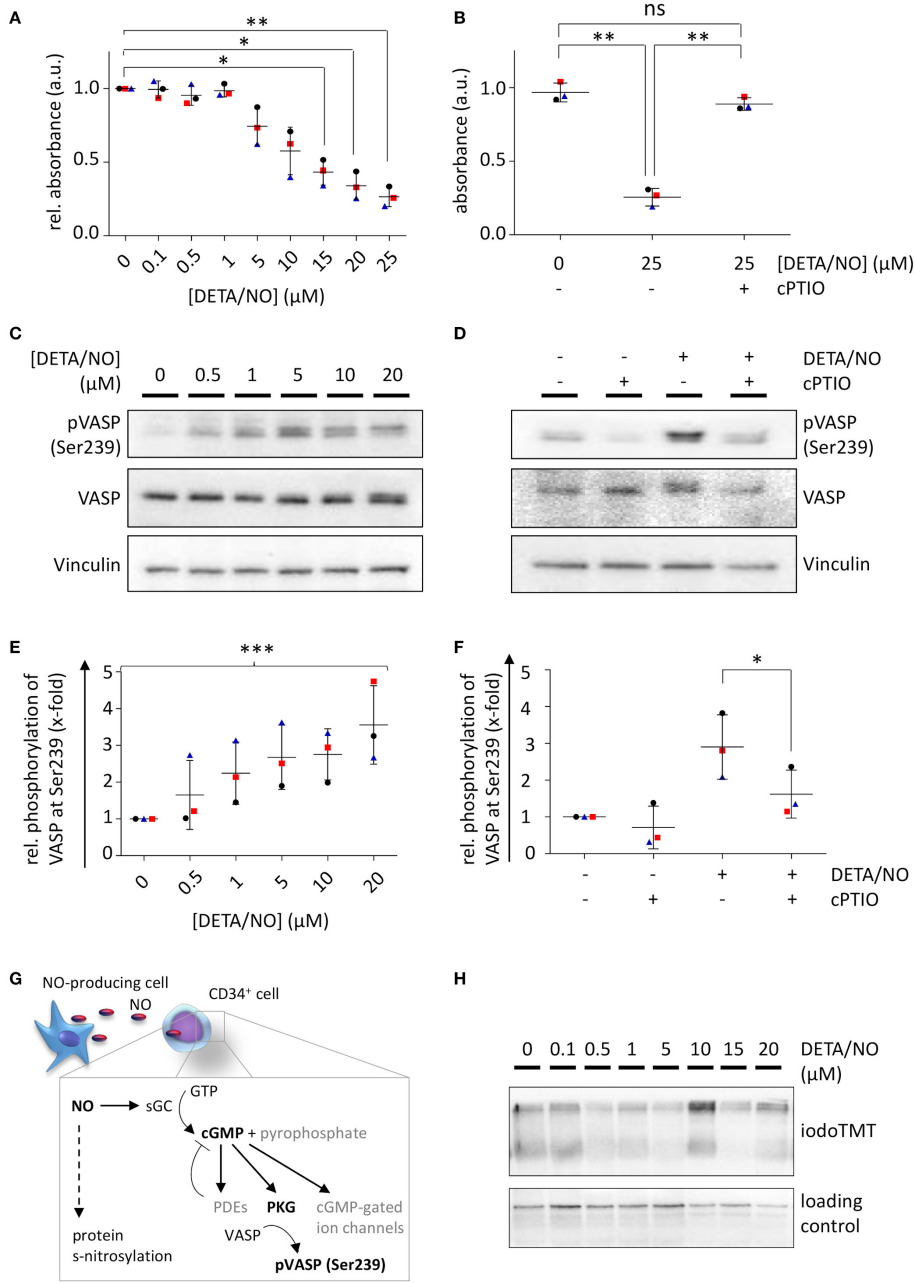
Nitric oxide (NO) signaling in human hematopoietic stem and progenitor cells (HSPCs) after *in vitro* culture with diethylenetriamine/nitric oxide adduct (DETA/NO). Activation of classical NO signaling was detected via intracellular cyclic guanosine monophosphate (cGMP) levels by a competitive enzyme-linked immunoassay. Substrate conversion was measured by absorbance and inversely correlates with intracellular cGMP levels. **(A)** Absorbance values relative to the control (y-axis) shown for up to 25 μM DETA/NO (x-axis). **(B)** Absorbance values (y-axis) after stimulation with 25 μM DETA/NO and/or preincubation with the NO scavenger cPTIO (x-axis). Classical NO signaling was further analyzed by vasodilator-stimulated phosphoprotein (VASP) phosphorylation at Ser239 as shown by a representative protein immunoblot after stimulation with **(C)** up to 20 μM DETA/NO and **(D)** 10 μM DETA/NO and/or after preincubation with cPTIO (Vinculin as a loading control). Gray values of VASP phosphorylation at Ser239 relative to the solvent control derived from protein immunoblots after stimulation of HSPCs with **(E)** up to 20 μM DETA/NO and **(F)** 10 μM DETA/NO and/or preincubation with DETA/NO. **(G)** Schematic illustration of NO-mediated signaling via the classical NO signaling pathway and s-nitrosylation. **(H)** Representative protein immunoblot of s-nitrosylation sites (marked by iodoTMT) after 5 days culture with up to 20 μM DETA/NO (Vinculin as loading control). Dot plots display the mean of *n* = 3 independent experiments with corresponding standard deviations as lines with error bars. Statistically significant intermean differences as per ANOVA (comparison of each column with the control) are indicated as follows: *P < 0.05; **P < 0.001. Curved brackets show linear trends with ***0.0001 < *P* < 0.001.

To check, whether the effect of NO on HSPC proliferation and differentiation was mediated by classical or nonclassical pathways, we monitored the activation of the classical NO signaling by two independent assays. The intracellular concentration of the second messenger cGMP was monitored via a competitive enzyme-linked immunoassay, where the analyte is subjected to analysis together with an enzyme-linked competitor antigen. Low intracellular concentrations of cGMP, therefore, cause high enzymatic conversion of the substrate, leading to high detected absorption values and *vice versa*. Treatment with up to 25 μM DETA/NO caused a decline in absorption beginning at 1 μM DETA/NO and thus revealed an increase in intracellular cGMP in HSPCs above this concentration (Figure 5A). Mean absorbance values were significantly reduced in comparison to the control at concentrations above 10 μM DETA/NO. Pretreatment of the cells with the stochiometric NO scavenger cPTIO reverted this effect, proving that the increase in cGMP was caused specifically by the release of NO (Figure 5B). To additionally monitor classical NO signaling, VASP phosphorylation by PKG at Ser239 was analyzed as a downstream signaling event. By treatment of HSPCs with increasing concentrations of DETA/NO, VASP phosphorylation at Ser239 increased significantly (Figures 5C,E). In addition, this phosphorylation event was seen at concentrations as low as 0.5 μM DETA/NO and thus at lower concentrations than indicated by competitive immunoassays. At low concentrations, specific phosphorylation of VASP at Ser239 was observed without phosphorylation at the second PKG phosphorylation site Ser157 (which causes an apparent shift in molecular weight when separated via SDS-PAGE). Pretreatment of HSPCs with cPTIO reduced the VASP phosphorylation at Ser239 significantly compared to the positive control (Figures 5D,F) and thus proved the specific phosphorylation of VASP downstream of NO. Beside classical signaling, NO can also act via posttranslational modification of protein thiols, termed s-nitrosylation (Figure 5G). Therefore, we analyzed the presence of these modification events by an indirect labeling method. Enhanced s-nitrosylation events were detected in HSPC lysates after treatment with high concentrations such as 10 μM of DETA/NO (data from representative donor shown in Figure 5H, additional data and quantification given in Supplementary Figure 6). Together, these findings lead to the conclusion that DETA/NO activates classical NO signaling at low concentrations in the employed DETA/NO concentration range but is only detectable at these low concentrations after sufficient signal amplification along the signaling pathway. High concentrations of DETA/NO additionally lead to the modification of protein thiols by nitrosylation, an event that is thought to function in nonclassical NO signaling.

## Discussion

NO is a central regulator of a variety of cellular processes at low concentrations, while elevated levels mediate toxic effects by unspecific protein and DNA modifications. Based on *in vitro* assays, physiologically relevant NO concentrations prevail below 50 nM, while toxic effects mediated by nitrosative stress were observed above 1 μM NO (Thomas et al., 2015). In contrast, human HSPCs were found to be more sensitive toward exogenous stimulation with NO in the present study. While concentrations up to 20 μM DETA/NO were well tolerated by HSPCs, higher concentrations induced toxic effects, indicating a threshold concentration of approximately 20 nM free NO in HSPCs according to a widely used approximation (Pervin et al., 2001, 2007). Thus, apoptosis processes seem to be regulated more tightly in human HSPCs than in cancer cell lines often employed in early *in vitro* studies (Thomas et al., 2004). As the induction of apoptosis is central to avoid the accumulation of DNA damage in the presence of high levels of reactive species (such as ROS and RNS) and thus to lower the chance of malign transformation, apoptosis processes are strictly regulated in stem cells (Biechonski et al., 2018). *In vivo*, the physiologically relevant concentration range of free NO is estimated to be between 100 pM and 5 nM (Hall and Garthwaite, 2009). Therefore, in the present study, concentrations from 0.1 up to 20 μM DETA/NO—corresponding to 0.1–20 nM free NO—were employed to monitor the full range of NO-mediated effects on human HSPC behavior both during homeostasis and under acute stress.

The role of NO as a regulator of cell proliferation has been thoroughly discussed in the literature both in physiological and pathological settings such as tumor development and angiogenesis (Ridnour et al., 2005, 2006, 2008; Villalobo, 2006; Napoli et al., 2013; Thomas et al., 2015). Although findings are often contradictory due to the variety of used test systems and employed NO concentration ranges, a consensus has been reached where low NO concentrations favor proliferation, whereas high NO concentrations mediate antiproliferative and toxic effects (Napoli et al., 2013). Nevertheless, there are exceptions to this rule depending on the monitored cell type. Regarding HSPCs, little information is available to date. Using NO scavengers *in vitro* (Kashiwakura et al., 1998) and pharmacological inhibitors for NO synthesis *in vivo* (Michurina et al., 2004), an antiproliferative effect of NO was demonstrated on murine HSPCs. Similar effects were observed using human bone marrow-derived CD34^+^ cells *in vitro* (Maciejewski et al., 1995). Earlier publications often overestimated physiological concentrations of NO and thus employed toxic NO concentrations. An essential role of NO for murine HSPC proliferation was found in NOS3^−/−^ mice (Aicher et al., 2003) and by exogenous stimulation of bone marrow-derived mononuclear cells (Krasnov et al., 2008). Further, NO mediated stress-induced expansion of HSCs in zebrafish (Hall et al., 2012) and acted as a pro-proliferative signaling cue in *in vitro* co-culture models with macrophages (Luo et al., 2018). Taken together, these publications argue for NO as a proliferative stimulus in HSPC biology and hint at NO as a supporter of HSPC self-renewal but are mainly based on results obtained from murine systems.

In the present study a wide concentration range of NO was chosen to allow monitoring the influence of NO on human HSPC proliferation in a concentration-dependent manner *in vitro*. Low concentrations of DETA/NO led to a highly reproducible increase in HSPC numbers in unstained cells and a donor-dependent increase in proliferation in CTV-stained cells supporting a pro-proliferative role of NO in human HSPC proliferation at low concentrations *in vitro*. Similar observations were made in murine HSPCs upon exogenous stimulation with a NO donor (Nogueira-Pedro et al., 2014). As low DETA/NO concentrations were activating classical NO signaling as observed via VASP phosphorylation at Ser239, an involvement of this pathway in the pro-proliferative response of HSPCs seems likely. This pathway also mediates the increased proliferation of murine neural stem cells upon exogenous stimulation with DETA/NO (Carreira et al., 2013). Little is known on the possible targets of pro-proliferative NO signaling under physiological conditions. Using cancer cell lines, cGMP-independent targets of NO signaling that are involved in cell cycle regulation and mitogenic signaling have been identified (Kumar et al., 2010; Batista et al., 2013); however, these findings might be difficult to transfer to primary cells from healthy donors, as regulatory proteins of the cell cycle are often deregulated in cancer cells (Diaz-Moralli et al., 2013). Thus, the low concentrations that were mediating pro-proliferative effects argue for the involvement of classical NO signaling, as s-nitrosylation events require higher concentrations of NO (Martinez-Ruiz et al., 2011). Consistently, s-nitrosylation events were solely observed when stimulating human HSPCs with increased concentrations of DETA/NO *in vitro*. At these elevated DETA/NO levels, human HSPC proliferation was slowed in the present study. This was, however, not attributed to NO-mediated toxic effects such as cell cycle arrest or induction of apoptosis, as HSPCs were actively cycling even at highest DETA/NO concentrations and showed vital morphology. Antiproliferative effects of NO were also observed during the differentiation of human peripheral blood-derived CD34^+^ cells into dendritic cells (Tiribuzi et al., 2013). Further, NO acts as a switch, which limits proliferation and induces differentiation in various types of stem cells (Beltran-Povea et al., 2015; Bonafe et al., 2015; Tapia-Limonchi et al., 2016) as well as during organ development in model organisms (Peunova et al., 2007). As CTV analysis additionally revealed the emergence of two HSPC subsets with distinct proliferative activities, an NO-induced change in HSPC differentiation seemed likely, as distinct differentiation states are associated with changes in proliferative activity (Passegue et al., 2005).

**Figure 6:**
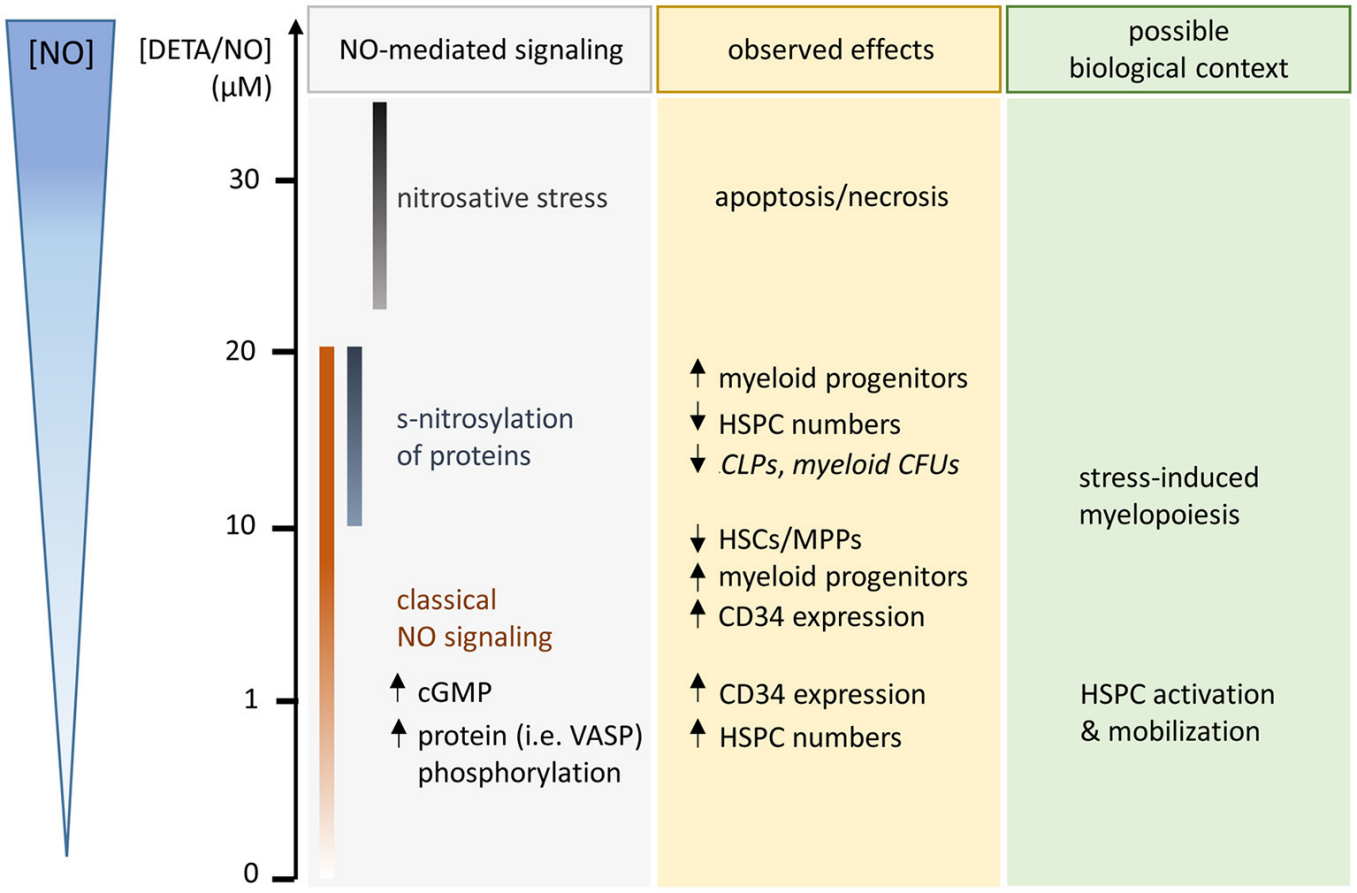
Schematic illustration of observed hematopoietic stem and progenitor cell (HSPC) responses after 5 days culture in the presence of the long-term NO donor diethylenetriamine/nitric oxide adduct (DETA/NO) *in vitro* (used concentrations and released levels of NO indicated by y-axis on the left side). NO-mediated signaling, which was detected in specific concentration ranges, is listed in the gray box with the concentration ranges illustrated by color-coded bars. Observed NO-mediated effects are specified in the yellow box (middle, statistically nonsignificant trends are displayed in italic), while a possible biological context for the observed effects is given in the green box (right side).

Via flow cytometry an NO-induced increase in CD34 expression was seen. This was, however, not caused by the NO-induced expansion of HSCs and MPPs, as exogenous stimulation with the gasotransmitter was not attributed to a prolonged retention of CD34 over more HSPC generations. Rather, both the CD34 expression level and the CD34^+^ cell fraction dropped at an earlier generation compared to the control. In addition, HSC and MPP cell fractions were reduced with increasing concentrations of DETA/NO. Based on these findings, exogenous stimulation of human HSPCs with NO acted as a cue for the differentiation into distinct HSPC subpopulations, which differ both in their CD34 expression level and in their proliferative activity. Similar observations were made in the literature by inhibition of NOS isoforms (Michurina et al., 2004). With rising DETA/NO levels, an increased myeloid differentiation was observed, which was at cost of CLPs at highest DETA/NO concentrations. Interestingly, CMP fractions were increased in highly proliferative subsets, while MEP fractions were amplified in a subset that underwent little cell divisions. As MEPs are highly CD34^+^ (Attar, 2014), they likely caused the increase in CD34^+^ cell fractions in early cell generations upon stimulation with DETA/NO, while the increase in CMPs and GMPs could have contributed to the NO-induced high CD34 expression in later cell generations. This is supported by the fact that HSPC subsets are characterized by distinct proliferative states with GMPs, CMPs, and MEPs showing highest, intermediate, and lowest proliferative activity, respectively (Passegue et al., 2005). In the murine system, NO-induced differentiation of HSPCs was observed frequently (Nogueira-Pedro et al., 2014; Jalnapurkar et al., 2016; Luo et al., 2018), and also in human CD34^+^ cells, cGMP analogs were shown to induce increased erythroid differentiation at the expense of early myeloid progenitors upon activation of classical NO signaling (Ikuta et al., 2016). Interestingly, observations via analysis of immunophenotypical markers were not mirrored completely in functional assays. Consistent with an NO-induced effect on all types of myeloid progenitors identified via flow cytometry, no single colony type was increased in CFU assays. Rather, a trend to increased myeloid colony numbers was observed at intermediate DETA/NO concentrations, and colony numbers seemed reduced at high DETA/NO concentrations (15–20 μM) for all myeloid colony types. These observations hint at the increase in functional myeloid progenitors at intermediate DETA/NO concentrations, while the increase in myeloid progenitors at high DETA/NO concentrations seemed to be correlated with either a loss of functionality *in vitro* or a later stage of myeloid differentiation resulting in reduced CFU numbers. At those high DETA/NO concentrations, increased levels of s-nitrosylation were detected. While low concentrations up to 5 μM DETA/NO were not correlated with increased s-nitrosylation, elevated levels of the modification were detected upon stimulation with 10 μM DETA/NO or above. These cGMP-independent modifications have been shown to mediate NO-induced increase in CD34 (Jalnapurkar et al., 2016) and CXCR4 expression (Zhang et al., 2007) on murine and human HSPCs, respectively. *In vivo*, these modifications are mediated by cellular mechanisms that ensure their specificity. In contrast, NO exhibits a diverse activity toward thiols *in vitro*, and modifications seem to occur in a less regulated manner (Wynia-Smith and Smith, 2017). As the increase in s-nitrosylation correlated with an increased myeloid differentiation of HSPCs, which was accompanied by a decreased potential to form myeloid colonies, concentrations above 10 μM DETA/NO could drive unspecific protein modifications *in vitro* and thus mimic unspecific s-nitrosylation as present under acute stress. As highest employed DETA/NO concentrations of 20 μM were close to toxic NO concentrations with beginning induction of apoptosis in some donors, we expect that effects seen at this highest concentration could have been partially mediated by apoptotic processes and could mimic acute stress responses. The present study identified several concentration ranges of NO, which induced a variety of cellular responses emphasizing the importance of used concentrations of the gasotransmitter (Figure 6). Contradictory findings in the literature are thus likely derived from the high variance of used NO concentration ranges and NO donors. Further, results obtained by the knockout or inhibition of NO synthesis do not allow for the concentration-dependent analysis of NO signaling.

The cellular redox state is linked to central HSPC behavior such as proliferation or differentiation (Ludin et al., 2014). ROS are, on the one hand, correlated with cell cycle arrest and apoptosis when critical concentrations are reached but are generally required for regular differentiation of HSCs (Ludin et al., 2014). Similar to the effects observed in the present study for NO, elevated concentrations of ROS are linked to increased myeloid differentiation. Due to their reactive nature, observed effects of ROS and NO-mediated effects overlap. The onset of differentiation, marked by a decrease in HSC and MPP fractions, was however already observed at intermediate DETA/NO concentrations where increased s-nitrosylation was not detected. Thus, classical NO signaling could also have contributed to this effect. Likewise, molecules of the classical NO-signaling pathway such as cGMP were shown to influence HSPC differentiation by the modulation of several transcription factors (Ikuta et al., 2016).

*In vivo* NO supports HSC retention, while elevated concentrations lead to HSPC expansion and mobilization (Chigaev et al., 2011; Nogueira-Pedro et al., 2014; Gur-Cohen et al., 2016). As the NO-induced mobilization of HSPCs is correlated with the modulation of VLA4 affinity *in vivo*, observed effects at intermediate to high concentrations of DETA/NO could mimic HSPC mobilization under acute stress. Together with the egress from the endosteal niche in the bone marrow to the vascular niche, HSCs that become metabolically active are prone to myeloid differentiation (Kopp et al., 2005). Neighboring niche elements such as endothelial cells, specialized stroma cells, as well as mature hematopoietic cells could serve as possible sources of targeted NO generation and stimulate HSPCs in a paracrine manner. Close to sinusoidal blood vessels, megakaryocytes are often found, which could be a potential source of NO and which are colocalized with myeloid-primed HSCs (Pinho et al., 2018). In addition to the regulation of HSCs via the CXC motif chemokine 4 (Pinho et al., 2018), NO could be a factor, which influences HSCs in a direct or indirect manner and, depending on the concentration and duration of the signal, mediates the NO-induced mobilization and differentiation of the cells. Similar to the murine system, NO could act as a switch in the human HSC niche to balance HSC retention in the niche and mobilization of HSPCs to the blood system.

## Conclusion/Summary

NO has been discussed as a central signaling molecule in cell proliferation and differentiation in a variety of cell types both *in vivo* and *in vitro*. Regarding HSPCs, no clear picture was available due to the variety of used cell types, employed concentration ranges of NO, or the large variation in regard to experimental design in the literature. Complicating matters further most studies were based on the murine system, which differs (at least in some aspects) from the human system in regard to NO synthesis. In the present study, we could demonstrate that the influence of NO on human HSPC proliferation and differentiation is highly dependent on its concentration. At low concentrations, NO induced a proliferative response of HSPCs, which might play a role *in vivo* in HSPC expansion and mobilization. This effect was likely based on classical NO signaling pathways, which were shown to be activated even at lowest used DETA/NO concentrations. At intermediate levels, NO slowed the proliferation of HSPCs and induced differentiation, which was in favor of early myeloid progenitors at highest concentration correlating with increased levels of s-nitrosylation. These responses might be relevant in stress-induced myelopoiesis where HSPCs differentiate to meet the increased demand of myeloid progenitors. Therefore, different concentrations of NO might be used to induce proliferation or differentiation in human HSPC culture *in vitro*. Thus, the present study paves the way for further evaluation of the potential of NO for controlled expansion or guided differentiation of human HSPCs *in vitro*, which would be beneficial for many cellular therapies.

## Data Availability Statement

The original contributions presented in the study are included in the article/Supplementary Materials, further inquiries can be directed to the corresponding author/s.

## Ethics Statement

Cord blood samples for the described studies were obtained after informed consent of the parents and approval by the local Ethics Committee (Ethik-Kommission der Landesärztekammer Baden-Württemberg, project number B-F-2013-111).

## Author Contributions

JH: conception and design, collection and assembly of data, data analysis and interpretation, manuscript writing, and final approval of the manuscript. SK: collection and assembly of data. KB: collection and assembly of data and administrative support. CL-T: conception and design, manuscript writing, and final approval of manuscript. All authors contributed to the article.

## Dedication

This paper is dedicated to the memory of our co-author and dear colleague Saskia Kraus, who passed away far too young and completely unexpectedly during the review period of this work.

## Conflict of Interest

The authors declare that the research was conducted in the absence of any commercial or financial relationships that could be construed as a potential conflict of interest.
