# Supplementary material for: Nitric Oxide in the Control of the in vitro Proliferation and Differentiation of Human Hematopoietic Stem and Progenitor Cells
Source: Front Cell Dev Biol. 2021 Feb 9;8:610369. doi: 10.3389/fcell.2020.610369 (PMC7900502; doi:10.3389/fcell.2020.610369)
Supplement: Supplementary file 1 [file Data_Sheet_1.pdf]

Supplementary Material

Nitric Oxide in the Control of the *in vitro* Proliferation and Differentiation of Human Hematopoietic Stem and Progenitor Cells

Julia Hümmer, Saskia Kraus, Katharina Brändle, Cornelia Lee-Thedieck*

Supplementary Figures

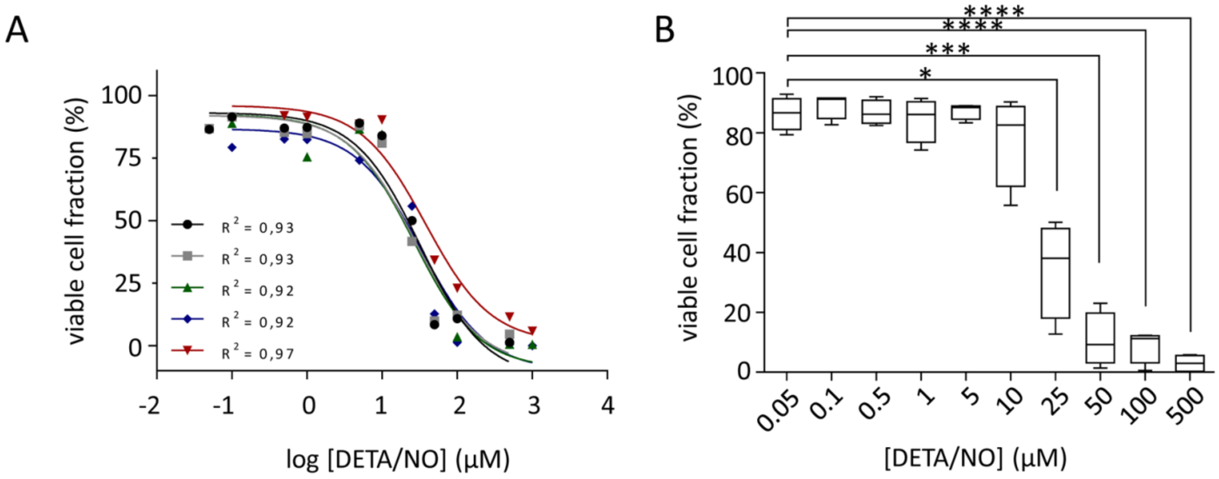

Supplementary Figure 1. Concentration dependent effect of DETA/NO on human HSPC viability after 5 days of culture *in vitro*. A) Viable cell fraction (Annexin V^- $Sytox^-$) (y-axis) after culture in presence of up to 500 µM DETA/NO shown against the decadic logarithm of the DETA/NO concentration (x-axis). Data points are interpolated by a hill function with the corresponding coefficients of determination (R^2) shown in the plot. B) Viable cell fraction (Annexin V^- $Sytox^-$) (y-axis) after *in vitro* culture with up to 500 µM DETA/NO (x-axis). Boxplot graph display shows the median as a line across the boxes of n = 5 independent experiments. Lower and upper boxes indicate the 25th to the 75th percentile. Whiskers represent the maximum and minimum values. Statistically significant inter-mean differences as per ANOVA (comparison of each column with a control column) are indicated as follows: * = $P < 0.05$; *** = $0.0001 < P < 0.001$; **** = $P < 0.0001$.

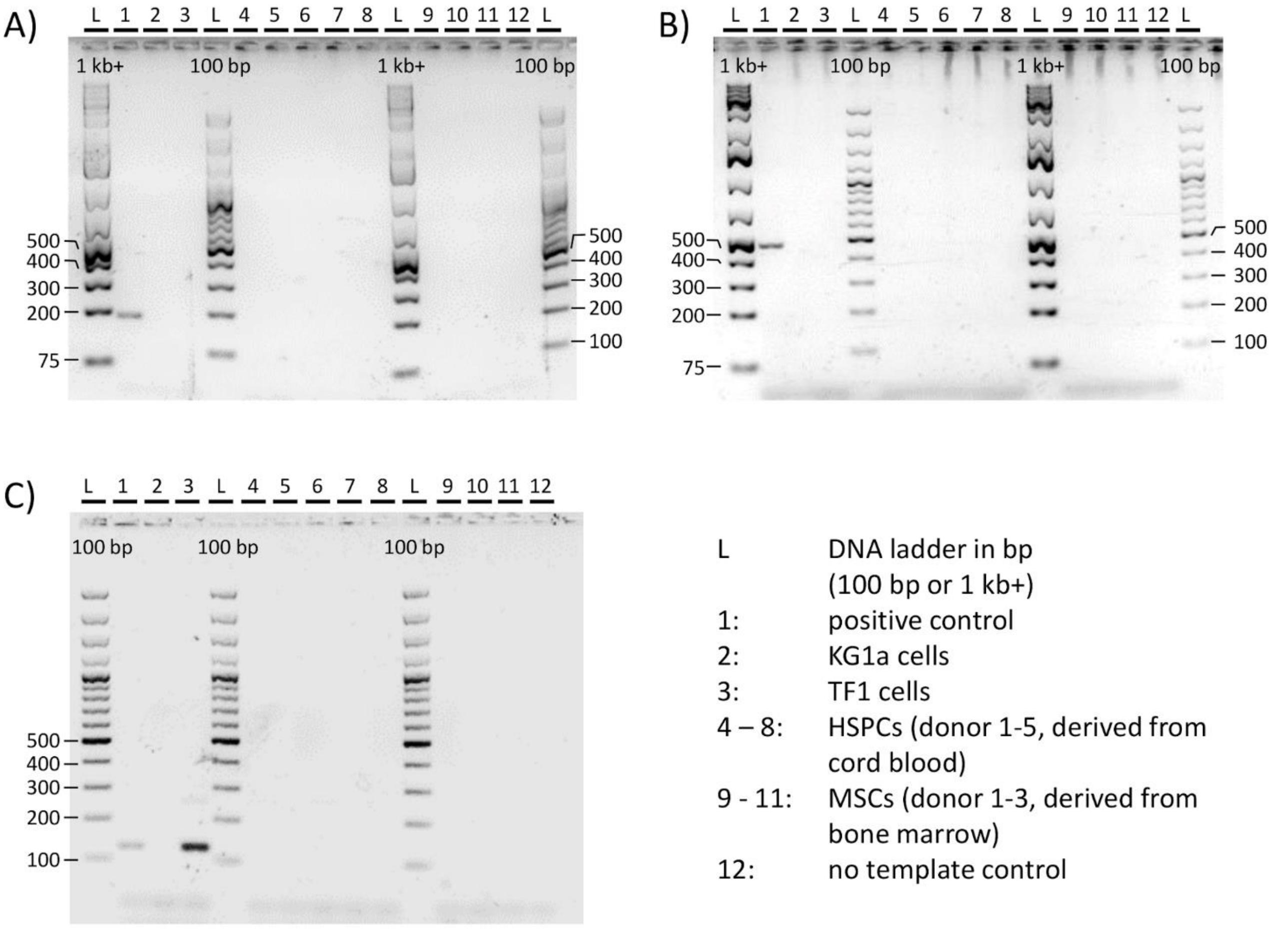

Supplementary Figure 2. Evaluation of nitric oxide synthase (NOS) expression profiles in hematopoietic cells and mesenchymal stromal cells (MSCs) via reverse-transcription polymerase chain reaction (RT-PCR) and agarose gel electrophoresis. As positive controls for the expression of A) NOS1, B) NOS2 and C) NOS3 the amplified PCR products from cDNA obtained from A) HaCat, B) Caco-2 and C) THP-1 cells were loaded into gel pocket 1. In lanes 2 and 3, RT-PCR products of the HSPC model cell lines KG1a and TF-1 were loaded, while lanes 4-8 contained PCR products from cDNA from HSPCs from five donors. RT-PCR products of bone-marrow derived MSCs were placed in lanes 9-11 and lanes 12 contained the no template control.

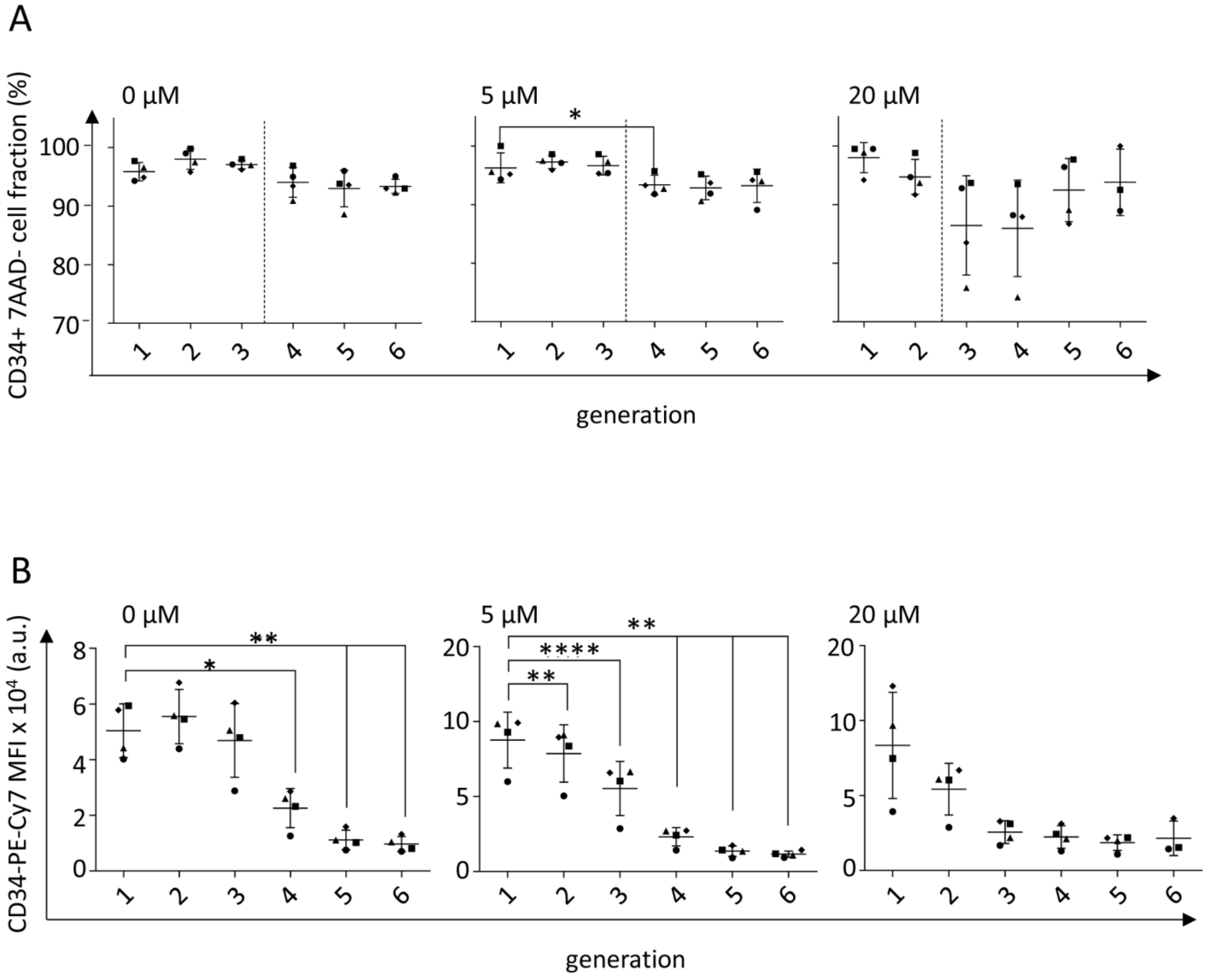

Supplementary Figure 3. Effect of exogenous stimulation with DETA/NO on CD34 expression of human HSPCs after 5 days of culture *in vitro*. A) CD34^{+} 7AAD^{-} cell fraction (y-axis) and B) mean fluorescence intensity signal in the CD34 channel (y-axis) depending on the cellular generation marking the number of undergone cell divisions (x-axis) after culture in presence of 0 µM, 5 µM and 20 µM DETA/NO. Dotplot graphs display the mean of n = 4 independent experiments with corresponding standard deviations as lines with error bars. Vertical dashed line indicating the drop in CD34 expression. Statistically significant inter-mean differences as per ANOVA (comparison of column means with the column mean of generation 1) are indicated as follows: * = $P < 0.05$; ** = $P < 0.001$, **** = $P < 0.0001$.

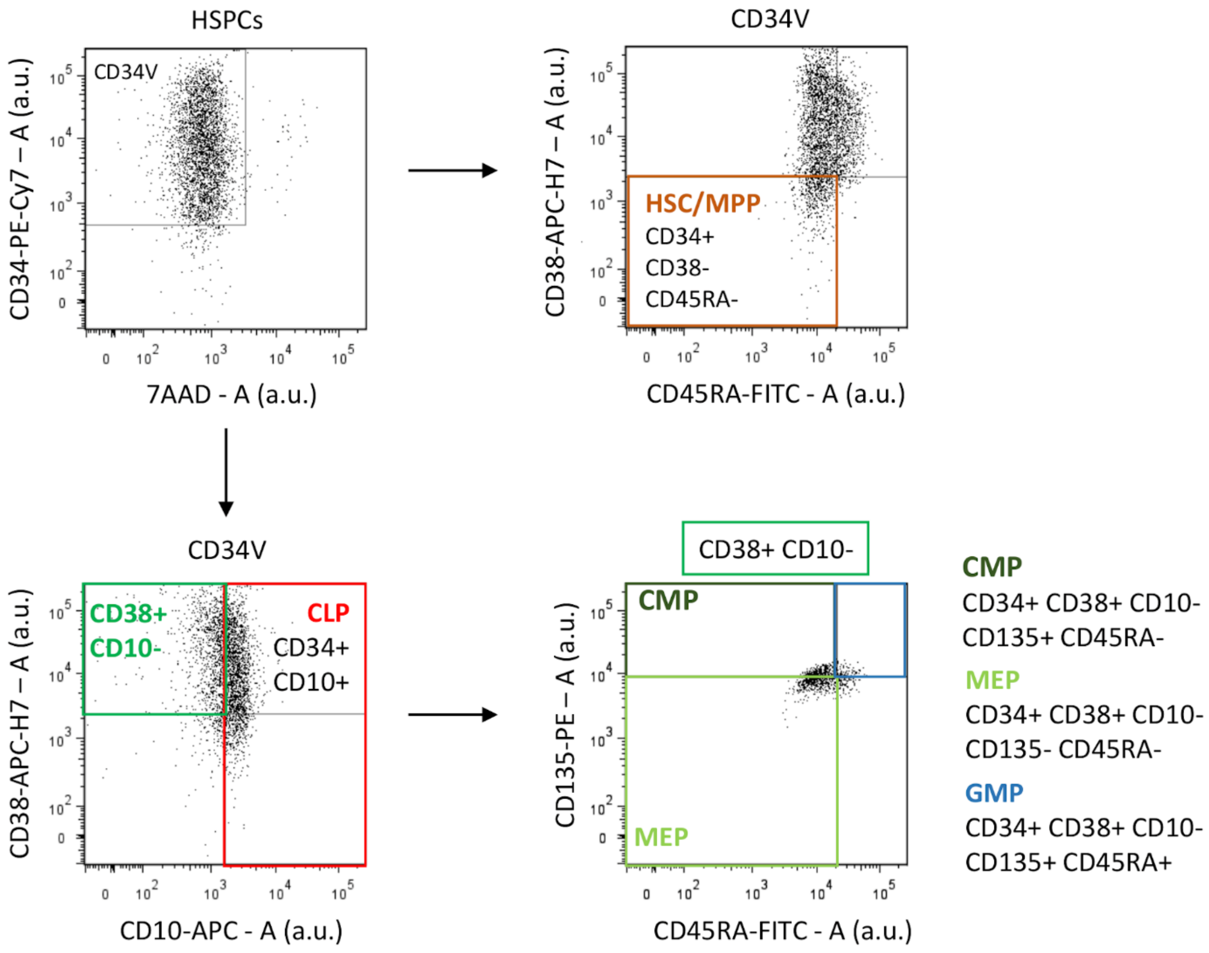

Supplementary Figure 4. Visualization of the gating strategy applied for flow cytometric analysis of HSPC subsets. In a first step HSPCs were gated for their expression of CD34 in the vital, 7AAD^{-} cell fraction. These vital CD34^{+} cells were further gated for their lack of CD38 and CD45RA expression yielding HSCs and MPPs or their additional expression of CD10 marking CLPs. Vital CD34^{+} cells that co-expressed CD38, but lacked CD10 expression were further sub-gated for their expression of CD45RA and CD135. CMPs were identified by their expression of CD135 but were CD45RA^{-}, while GMPs were co-expressing both markers. MEPs were lacking both CD135 and CD45RA expression.

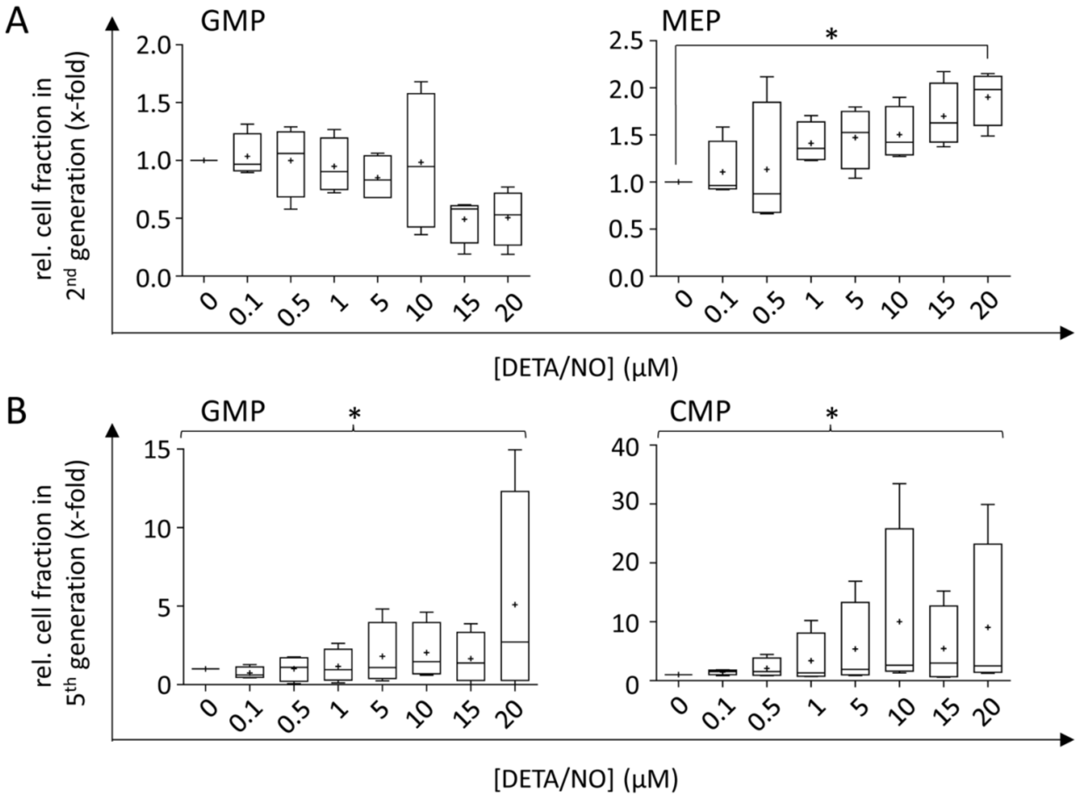

Supplementary Figure 5. Concentration-dependent effect of up to 20 µM DETA/NO on myeloid differentiation of human HSPCs *in vitro*. Myeloid progenitor cell fractions relative to the solvent control(y-axis) in generation A) 2 and B) 5 as determined immunophenotypically by flow cytometry. On the left side GMP cell fractions are displayed while on the right side A) MEP and B) CMP fractions are shown. Boxplot graphs display the median as a line across the boxes of n = 4 independent experiments with the mean shown as a plus. Lower and upper boxes indicate the 25th to the 75th percentile. Whiskers represent maximum and minimum values. Statistically significant inter-mean differences as per ANOVA (comparison of each column with the control) are indicated as follows: * = $P < 0.05$. Curved brackets indicate linear trend with: * = $P < 0.05$.

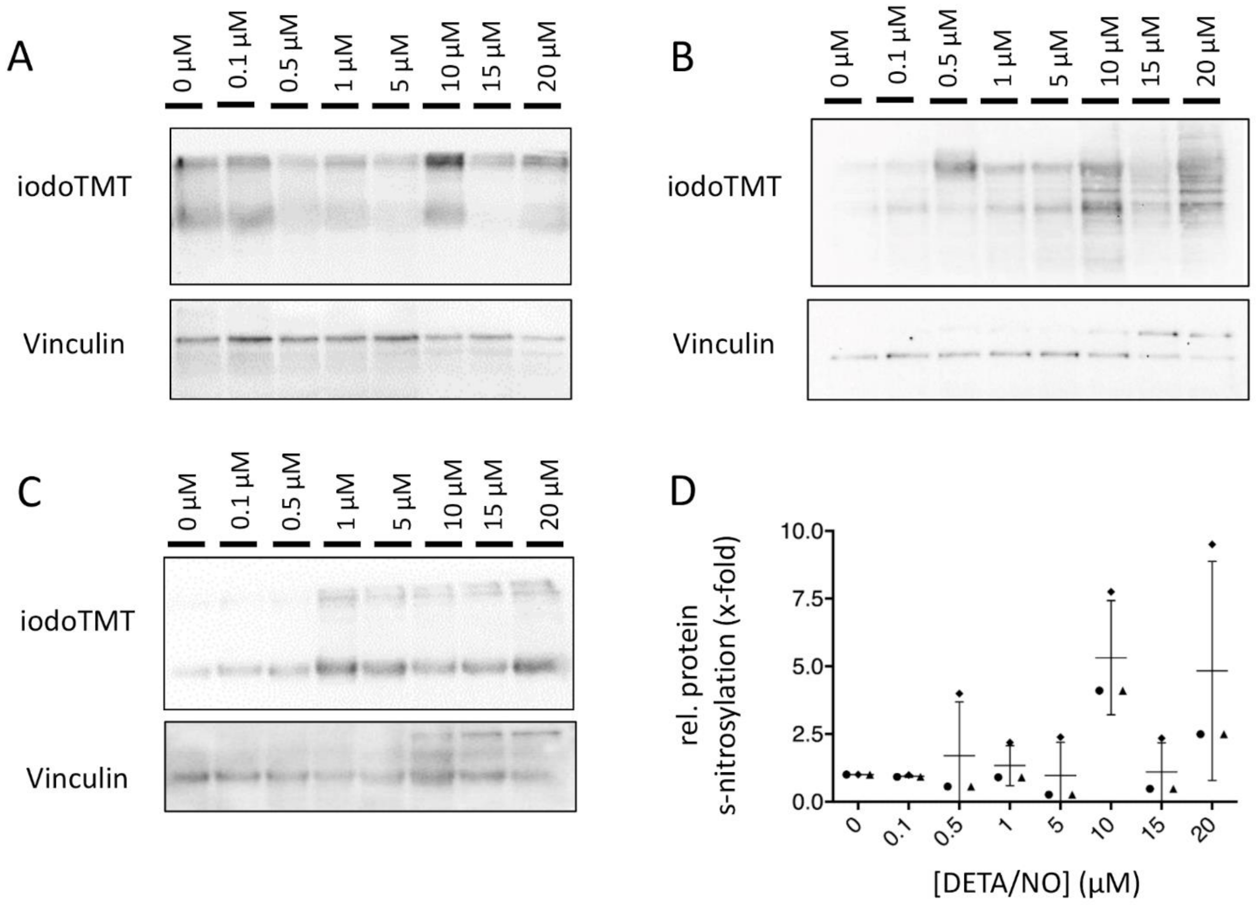

Supplementary Figure 6. Concentration-dependent effect of up to 20 µM DETA/NO on protein s-nitrosylation events in human HSPCs *in vitro*. A) - C) Protein immunoblots of s-nitrosylation sites (marked by iodoTMT) after 5 days of culture with up to 20 µM DETA/NO (Vinculin as loading control) for n = 3 donors. D) Grey values of iodoTMT signal to the solvent control derived from protein-immunoblots after stimulation of HSPCs with up to 20 µM DETA/NO. Dot plot displays the mean of n = 3 independent experiments with corresponding standard deviations as lines with error bars.
